# Memory-making interventions for children with life-threatening or life-limiting conditions and their families: A systematic review of evidence and implications for practice

**DOI:** 10.1177/02692163251353006

**Published:** 2025-07-25

**Authors:** Razieh Safarifard, Gemma Kiernan, Yvonne Corcoran, Eileen Courtney, John Mitchell, Terrah Akard, Veronica Lambert

**Affiliations:** 1 School of Nursing, Psychotherapy and Community Health, Faculty of Science and Health, Dublin City University, Glasnevin Campus, Dublin, Ireland; 2 Head of Partnerships, Barretstown Children’s Charity, Barretstown Castle, Ballymore Eustace, Co Kildare, Ireland; 3 School of Nursing, Vanderbilt University, Nashville, TN, USA

**Keywords:** Paediatric, palliative care, memory-making, family support, bereavement care

## Abstract

**Background:**

Emotional and psychosocial support is vital for children with life-limiting or life-threatening conditions and their families. Memory-making interventions, which create lasting memories, are gaining recognition, yet a comprehensive synthesis of their efficacy and scope is lacking.

**Aim:**

To systematically review and synthesize evidence on memory-making interventions for children and young people aged 0–19 years with life-threatening or life-limiting conditions and their families in paediatric palliative and bereavement contexts.

**Design:**

A systematic review conducted in accordance with Joanna Briggs Institute guidance for mixed-methods reviews and reported using PRISMA guidelines. Narrative synthesis was used to identify key themes related to the effectiveness, implementation and family experiences of these interventions.

**Data sources:**

PubMed, EMBASE, CINAHL, PsycINFO, Web of Science, Cochrane Library and Scopus.

**Results:**

Eleven articles met the inclusion criteria, identifying three categories of memory-making interventions: storytelling-based, art-based legacy and physical keepsake creations. While statistical significance was limited, studies suggested small to moderate psychosocial benefits. Narrative synthesis identified four key themes: emotional expression and comfort; family connection and communication; memory preservation and personalization; and grieving support and continued bonds. Digital storytelling was the most common intervention. Families emphasized the need for personalized and culturally sensitive approaches to improve engagement and relevance.

**Conclusions:**

Memory-making interventions provide valuable emotional and psychosocial benefits for children and young people and their families in paediatric palliative and bereavement contexts. Tailored, well-supported interventions can strengthen resilience and well-being. Addressing challenges like technological barriers and cultural sensitivities may optimize these interventions and improve care quality.

**What is already known about the topic?**

- Memory-making interventions are increasingly recognized in paediatric palliative and bereavement care as strategies that support psychosocial well-being by helping families create lasting connections and fostering emotional expression.
- Previous studies have examined various forms of memory-making interventions in healthcare contexts, such as legacy-building and digital storytelling.
- Consensus on the effectiveness and implementation of memory-making interventions specifically for children and young people with life-limiting or life-threatening conditions is limited.

**What this paper adds**

- Findings suggest that successful implementation of memory-making interventions relied on tailored approaches that consider individual children and family needs, cultural sensitivity and the availability of trained facilitators, such as healthcare professionals, paediatric palliative care specialists and creative therapists (e.g. art and music therapists).
- This review highlighted the critical importance of flexibility and responsiveness in delivering memory-making interventions.
- Despite positive feedback from families, factors such as technological accessibility, cultural misalignment and inadequate support can hinder the uptake and impact of memory-making interventions.

**Implications for practice, theory or policy**

- Creating a space for memory-making interventions is important for enhancing family cohesion, emotional regulation and continued bonds in bereavement care.
- A standardized yet adaptable approach to guide healthcare teams in implementing memory-making interventions is important to address cultural norms and personalization to the unique needs of each family.
- There is a need for further research to establish culturally sensitive and more personalized memory-making interventions tailored to the needs of children, young people and their families in paediatric palliative and bereavement contexts.

## Introduction

Memory-making interventions, often referred to as legacy building, have become increasingly recognized as integral psychosocial approaches in paediatric palliative and bereavement care. Interventions such as memory boxes, hand and footprint jewellery and moulds help families document their child’s life and maintain meaningful connections in end of life care and after their child’s death, supporting the grieving process.^1^ In line with this, the National Institute for Health and Care Excellence (NICE) Guideline NG61 on end of life care for infants, children and young people with life-limiting conditions recommends that, as a child nears end of life, professionals support families in engaging in meaningful rituals and preserving memories, such as taking photographs, hair locks, or handprints.^2^

Given the estimated 21 million children and young people worldwide affected annually by life-limiting and life-threatening conditions, with approximately 8 million requiring specialized paediatric palliative care,^3,4^ there is a growing need for comprehensive, multidisciplinary approaches, including memory-making interventions.^5,6^ Life-limiting conditions refer to illnesses or disorders for which no realistic prospect of cure exists, often resulting in early death, whereas life-threatening conditions are associated with a significant risk of death but may respond to curative or life-prolonging treatment.^7,8^ Support for children and young people in these circumstances requires coordinated care that integrates physical, psychological, social and spiritual dimensions.^9–11^ Tailoring end-of-life and bereavement care for families is also recognized as a critical element of high-quality, holistic paediatric palliative care.^12–14^ Memory-making interventions can fulfil this need by helping families create lasting memories and maintain a bond with their child, offering comfort and meaning during end-of-life care and bereavement.^14–19^

The emotional burden on parents can be overwhelming, particularly during the end-of-life phase, where anticipatory grief is common and following the death of the child.^20,21^ The death of a child profoundly impacts grieving parents, often resulting in significant adverse outcomes such as depression, anxiety and post-traumatic stress disorder.^22^ Addressing parental regret is particularly important, as unresolved feelings of regret may intensify grief and complicated bereavement outcomes.^21^ Parents who felt excluded or minimally involved in their infant’s care have reported ongoing guilt and remorse following their child’s death.^23^ Comprehensive support that addresses the emotional, psychological and practical challenges families face is essential, with a strong emphasis on family-centred psychosocial interventions to support overall well-being.^24–26^ Memory-making interventions are also associated with adaptive processes such as sense-making and benefit-finding, which support families in coping with loss and navigating complex grief.^19^ Despite increasing recognition of memory-making interventions in paediatric palliative and bereavement care, notable knowledge gaps remain about their psychosocial impacts. While studies suggest that memory-making interventions can support emotional well-being and family communication,^1,15,18^ comprehensive evidence detailing their benefits, the experiences of participating families and the factors affecting implementation is limited. Specifically, there is limited synthesis of findings related to the experiences of participating families.

Although previous reviews have examined memory-making and legacy interventions in palliative and bereavement care, most have either focussed on adult populations or synthesized experiences that occur after a child’s death.^27–30^ For instance, one review highlighted the general benefits of legacy interventions across adult populations, reporting improvements in emotional well-being and reductions in depressive symptoms.^27^ Another review focussed more broadly on adult paediatric healthcare, describing legacy creation as a collaborative process that reflects the unique identity and essence of the child.^28^ One qualitative thematic synthesis examined memory-making practices in critical care settings, drawing from both adult and paediatric contexts, yet offering limited focus on paediatric palliative care specifically.^29^ Another review centred on bereaved parents’ experiences, highlighting the importance of memory-making in grief processing while also identifying systemic barriers such as insufficient professional support and the lack of culturally sensitive practices.^30^

Current gaps in literature highlight the need to conduct a synthesis of evidence on memory-making intervention for children with life-limiting or life-threatening conditions and their families within paediatric palliative and bereavement care. This systematic review aims to offer critical insights into the effectiveness, implementation challenges and the psychosocial impact of these interventions.

## Aim

This study aimed to systematically review and synthesize evidence on memory-making interventions for children and young people (aged 0–19 years) with life-threatening or life-limiting conditions and their families in paediatric palliative and bereavement contexts. The objectives were to:

1. Identify and summarize the types of memory-making interventions used.
2. Assess the effectiveness of these interventions on psychosocial outcomes.
3. Explore the experiences of families participating in these interventions.
4. Examine the barriers and enablers to the successful implementation of these interventions.

## Methods

### Study design

This systematic review was conducted in accordance with the Joanna Briggs Institute (JBI) guidance for mixed-methods systematic reviews,^31^ and is reported following the Preferred Reporting Items for Systematic Reviews and Meta-Analyses (PRISMA) guidelines.^32^ See Supplemental File 1. Our protocol was registered in PROSPERO (CRD42024521388) and published in line with the PRISMA-P guidelines.^33^

### Search strategy and eligibility criteria

The comprehensive search strategy involved sourcing eligible studies from the following electronic databases: PubMed, EMBASE, CINAHL (EBSCO), PsycINFO (EBSCO), Web of Science, Cochrane Library and Scopus. Search dates were from January 1, 1985, to February 27, 2024. A combination of free text terms and controlled vocabulary adapted to each database were used related to ‘memory-making’, ‘legacy’, ‘paediatric’, ‘bereavement’, ‘child’, ‘family’ and ‘intervention’ (Supplemental File 2). Additionally, reference lists of identified reviews and seminal articles were also manually searched to capture additional studies not identified through database searches. The pre-specified inclusion and exclusion criteria for the review are shown in Table 1.

**Table 1.** Inclusion and exclusion criteria.

Eligibility criteria	Inclusion criteria	Exclusion criteria
Population	Children and young people aged 0–19 years with life-limiting or life-threatening conditions, along with their family members (e.g., parents, siblings, or others as identified in the included studies), as well as bereaved families.	Studies focussing on adults or patients without life-limiting or life-threatening conditions.
Interventions	Studies examining memory-making interventions such as the creation of physical mementoes, digital storytelling and participatory art projects.	Studies focussing solely on interventions not related to memory-making (e.g., medical treatments, pharmacological interventions).
Comparators	Usual care practices or other active interventions as defined by included studies. In qualitative or exploratory studies without direct comparators, no control group was required.	-
Outcomes	Primary outcomes: Quality of life, mental health indicators (stress, anxiety, depression), family communication. Secondary outcomes: Resilience, satisfaction with intervention, role of interventions in preserving memories and grief processes.	Studies not assessing psychosocial impacts or where the primary focus is not on the defined outcomes.
Study designs	Qualitative, quantitative and mixed-methods studies, including randomized controlled trials, cohort studies, case-control studies and qualitative studies.	Review papers, conference abstracts, thesis, editorials, commentary and case studies.
Publication years	Peer-reviewed journal articles published from 1985 to February 27, 2024.	-
Language	English.	Studies published in languages other than English.

### Data screening

**Figure 1.**
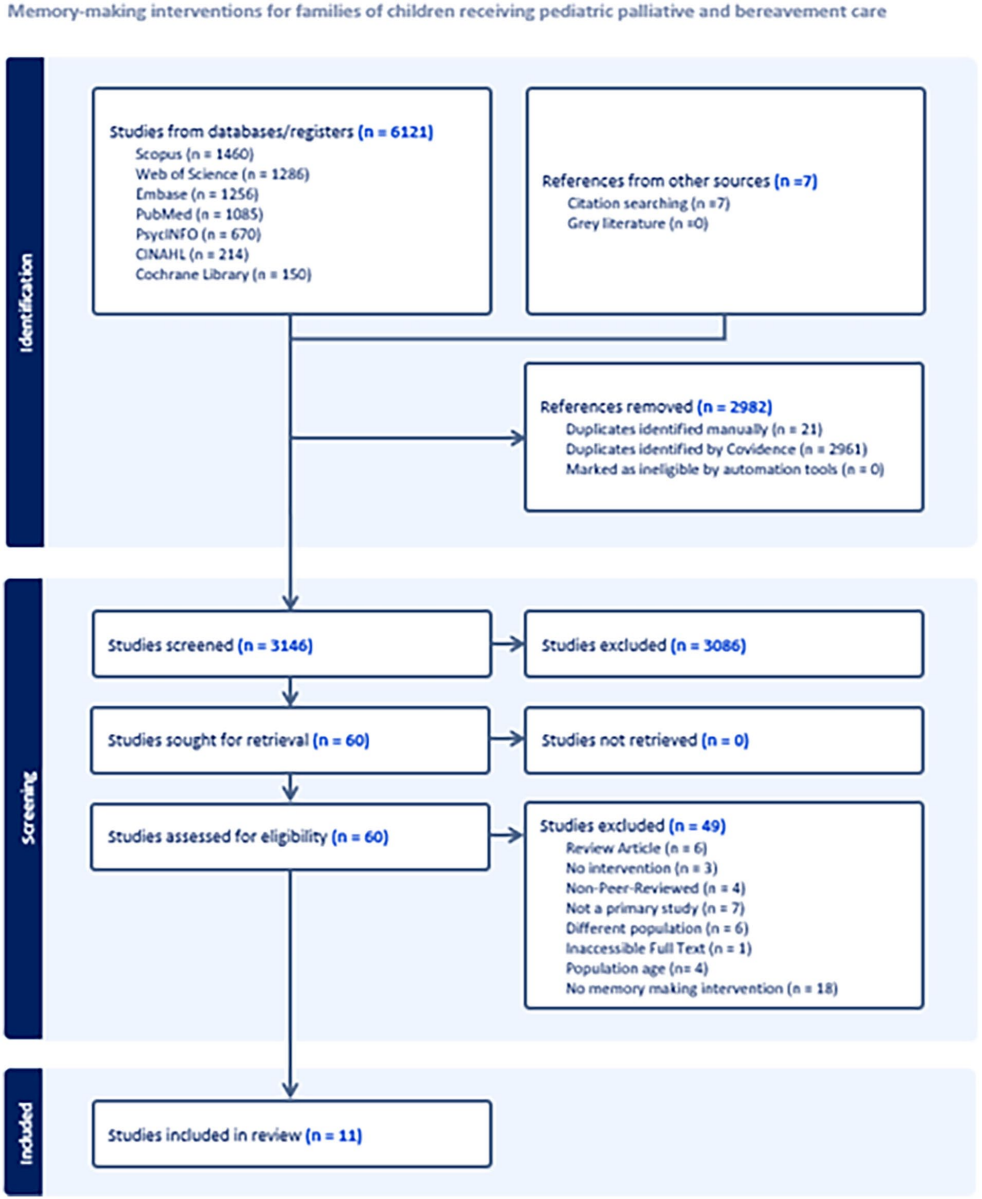
PRISMA flow diagram.

Search results were uploaded to Covidence software to remove duplicates and facilitate a two-stage screening process.^34^ In stage one, two reviewers (RS, VL) independently screened titles and abstracts against eligibility criteria, with a third reviewer (EC) resolving disagreements. In stage two, full-text screening followed a similar approach, with two independent reviewers (RS, VL) and a third reviewer (EC) resolving discrepancies. Reasons for exclusion were recorded (see Figure 1).

### Data extraction

Data were extracted by the first author (RS) using pre-designed, and piloted, forms for each study type (quantitative, qualitative, mixed methods). Extracted data included authors, title, year, country, study aims, design, participant demographics, interventions, comparators, outcome measures and key findings. Qualitative data on intervention components, using the Template for Intervention Description and Replication checklist,^35^ and potential barriers and enablers to implementing the interventions were also extracted. Extracted data were cross-checked for accuracy and completeness by a second reviewer (VL).

### Quality appraisal

Quality appraisal was conducted by two independent reviewers (RS, YC) using the mixed methods appraisal tool (MMAT). This tool assessed studies based on criteria specific to their design, including clarity of research questions, appropriateness of methods and coherence of data collection and interpretation.^36^ Studies were assessed using these criteria, ensuring a rigorous evaluation of their methodological quality. A third reviewer (VL) resolved any discrepancies. Studies were not excluded based on results of the quality appraisal; rather the purpose of the quality appraisal was to describe the quality of included studies to aid interpretation of the evidence.

### Data synthesis

We followed the Joanna Briggs Institute data synthesis guidelines for mixed methods systematic reviews.^34^ While a meta-analysis was initially planned, it was not feasible due to heterogeneity in study designs, outcomes and measures, as well as a lack of statistical comparability. As a result, a narrative synthesis was undertaken, which involved systematically organizing, describing and interpreting the study findings to identify and explore patterns across the included articles.^37^ Specifically, we employed open coding to categorize data into meaningful themes related to effectiveness, implementation and family experiences of memory-making interventions. These preliminary themes were further refined through discussion among reviewers to enhance analytical rigour and reliability. Patterns, differences and unique insights across studies were identified and synthesized narratively, capturing the nuanced experiences reported by families.

The template for intervention description and replication checklist was used to guide data extraction, ensuring a comprehensive and detailed understanding of each intervention’s components, delivery methods and participant engagement.^35^ This informed our synthesis and facilitated a thorough analysis of the interventions’ implementation and effectiveness.

## Results

Details of the review search process and results can be found in Figure 1.

### Study characteristics

The 11 studies included were published between 2015 and 2023, with 10 conducted in the United States^16–18,24,38–43^ and one in Ireland.^1^ The studies comprised four qualitative studies^1,24,41,42^ five randomized controlled trials^16–18,38,39^ including one feasibility and acceptability study^39^; and one non-randomized study.^40^ Sample sizes in individual studies ranged from 6 to 150 participants.

Six articles focussed on children and young people,^16–18,38,39,43^ while five included bereaved parents.^1,24,40 –42^ The age range of child participants was broad, from 3 days to 19 years, though most studies focussed on children aged 7–17 years. Cancer was the predominant diagnosis across eight studies, with other studies addressed various life-limiting conditions like neurodegenerative disorders. The interventions were applied in diverse settings, including neonatal intensive care units, hospitals, hospices, homes and web-based platforms, demonstrating the flexibility of memory-making interventions. Notably, four of the papers^16–18,43^ were derived from a single large-scale study but examined different aspects within that study. For a comprehensive overview of the methodology, participant demographics and outcomes, please refer to Table 2 and see additional details in Supplemental File 3.

**Table 2.** Summary of included studies.

Author (year)	Country	Study design	Intervention type	Participants (sample size)	Age (mean, range)	Setting	Context	Main outcome	MMAT score
Akard et al.^38^ (2015)	USA	Quantitative RCT	Digital storytelling intervention	Children with cancer (*n* = 28)	11, 7–17	Home	Paediatric palliative care	Feasibility of digital storytelling; preliminary trends towards improved emotional and school functioning and quality of life (not statistically significant)	5/5
Akard et al.^24^ (2018)	USA	Qualitative	Digital storytelling legacy-making	Bereaved parents (*n* = 6)	29.5 (parents)	NICU	Bereavement care	Parents reported digital storytelling as acceptable and beneficial for grief processing	5/5
Akard et al.^39^ (2020)	USA	Quantitative RCT- feasibility	Web-based legacy intervention	Children with relapsed/refractory cancer and their primary parent caregivers (*n* = 81)	10, 7–17 (children)	Web-based platform	Paediatric palliative care	High engagement and feasibility of web-based intervention	5/5
Andrews et al.^40^ (2020)	USA	Quantitative Non-RCT	Music therapy heart sounds	Bereaved parents of children (12)	7 (children)	Children’s hospital	Paediatric palliative care	Memory-making supported grieving and memory preservation	4/5
Schaefer et al.^41^ (2020)	USA	Qualitative	Legacy artwork	Bereaved parents (*n* = 12)	41.17 (parents)	Children’s hospital	Bereavement care	Legacy art provided emotional healing and enduring connection with deceased children	5/5
Akard et al.^16^ (2021)	USA	Quantitative RCT	Web-based legacy intervention	Children with advanced cancer and their parents (150)	10, 7–17 (children)	Web-based platform	Paediatric palliative care	Improved parent–child communication, especially with fathers (not statistically significant)	5/5
Foster Akard et al.^17^ (2021)	USA	Quantitative RCT	Web-based legacy intervention	Parents of children with advanced cancer (150)	10, 7–17 (children)	Web-based platform	Paediatric palliative care	Improved parental coping, small trends towards increased primary control and disengagement coping (not statistically significant)	5/5
Akard et al.^18^ (2021)	USA	Quantitative RCT	Web-based legacy intervention	Children with advanced cancer and their parents (150)	10, 7–17 (children)	Web-based platform	Paediatric palliative care	Improvements in quality of life; small effects in procedural anxiety and perceived physical appearance (not statistically significant)	5/5
Clarke and Connolly^1^ (2021)	Ireland	Qualitative	Legacy artwork	Bereaved parents (*n* = 6)	3 days-10 years (children)	Children’s hospice	Bereavement care	Memory-making supported anticipatory grief and continued bonds	5/5
Walden et al.^42^ (2021)	USA	Qualitative	Heart beat recordings (HBR)	Parents of children with PNDI (*n* = 11)	20–68 (parents)	Academic paediatric hospital	Bereavement care	Heartbeat recordings viewed as spiritual, comforting and essential to legacy creation	4/5
Cho et al.^43^ (2023)	USA	Quantitative RCT	Digital storytelling legacy-making	Children with recurrent/refractory cancer (150)	10, 7–17 (children)	Web-based platform	Paediatric palliative care	Trends towards enhanced communication and coping (not statistically significant)	5/5

Details on intervention rationale, procedures, materials and implementation were provided in the Template for Intervention Description and Replication checklist in Supplemental File 4. This included the intervention’s name, description, rationale, materials, procedures and delivery. For example, digital storytelling interventions were personalized and web-based for flexible engagement, art-based interventions involved creative activities led by trained therapists, and physical keepsakes used tangible materials cherished by families. This checklist ensured thorough documentation, supporting replication and understanding of these memory-making interventions.

### Quality of the evidence

The methodological quality of the 11 included studies was generally high, as per the mixed methods appraisal tool (MMAT) criteria, but some limitations were identified. Akard et al. focussed on feasibility and acceptability, limited generalizability and encountered user interface issues affecting retention.^39^ Andrews et al. had a small sample size and used retrospective self-reporting, raising the risk of recall bias.^40^ Walden et al. used a homogenous, purposefully selected sample, which may lead to selection bias.^42^ Overall assessments are available in Supplemental File 5.

#### Overview of types of memory-making interventions

We identified three main categories of memory-making interventions (i) storytelling-based interventions, (ii) art-based legacy interventions and (iii) physical keepsake creations. Storytelling, particularly digital storytelling, emerged as the predominant approach, employed in seven of the included papers.^16–18,24,38,39,43^ These storytelling interventions included a web-based platform where children or parents could create digital stories incorporating videos, photographs, music and personal stories.

Three papers reported on art-based legacy interventions. Two of these studies used legacy artwork^1,41^ involving various forms of creative expression such as hand/foot moulds, memory books and painting projects. Facilitated by art therapists, these interventions allowed for the creation of paintings and other creative expressions that included personal and meaningful elements like handprints or favourite colours. The third paper in the legacy artwork intervention category involved therapeutic approaches using music or sound, including the Music Therapy Heart Sounds programme.^40^

In the physical keepsake creations category, one study encompassed interventions creating physical items, such as fingerprint charms, handprint plates and heartbeat songs, which serve as enduring mementoes of the child’s life.^42^

#### Outcomes of memory-making interventions

Outcomes of memory-making interventions for children with life-limiting or life-threatening conditions and their families were assessed across seven quantitative studies.^16–18,38 –40,43^ Outcomes included quality of life, communication, coping strategies, acceptability and feasibility and other aspects of psychosocial functioning.

*Quality of Life*: Two studies assessed paediatric quality of life as an outcome.^18,38^ One study observed slight improvements in emotional and school functioning, with parents noting positive impacts on emotional comfort and family communication.^38^ Another study revealed modest improvements in procedural anxiety and perceived physical appearance.^18^ Findings of both studies did not reach statistical significance, and thereby the evidence on effectiveness remains inconclusive.

*Communication*: Akard et al.^16^ explored the impact on parent–child communication, noting improvements in communication quality, particularly between fathers and children. While these changes were not statistically significant, the findings suggested that memory-making interventions may help in reducing emotional barriers and enhancing communication dynamics within families.

*Coping strategies*: Akard et al. and Cho et al.^17,43^ investigated coping strategies, revealing trends towards increased use of primary control and disengagement coping mechanisms among parents, although findings were not statistically significant. These studies indicate potential benefits in enhancing adaptive coping, but further research is needed for conclusive evidence.

*Acceptability and feasibility*: Akard et al.^39^ investigated the acceptability and feasibility of a web-based legacy intervention for children with cancer. Families provided positive feedback, particularly regarding the usability of the intervention and the emotional support it offered. While the study did not quantify effectiveness, the qualitative impact was evident, with families highlighting how legacy activities, such as storytelling, fostered emotional expression and communication. This made the intervention both meaningful and acceptable, reinforcing its feasibility as a supportive tool.

*Other psychosocial functioning*: Andrews et al.^40^ demonstrated that memory-making interventions like the Music Therapy Heart Sounds programme can support emotional processing and provide comfort to families. These interventions have been well-received, with participants reporting high levels of satisfaction and perceived value.

#### Experiences of families participating in memory-making interventions

All 11 included articles reported data on family experiences of participating in memory-making interventions. Four studies were qualitative in nature,^1,24,41,42^ while six quantitative studies^16–18,38,40,43^ and one feasibility study^39^ included qualitative feedback on family experiences of memory-making interventions. The perspectives represented in these studies primarily include parents with data gathered through semi-structured interviews, reports, surveys and follow-up questionnaires.

The evidence on family experiences of engaging in memory-making interventions highlighted their important influence on psychological well-being and interpersonal dynamics during difficult periods. Data from included studies indicated that families treasure the comfort and connection memory-making interventions offer. The key themes from the narrative synthesis include:

##### Emotional expression, comfort and healing

Almost all of the included studies mentioned that memory-making interventions enhance emotional expression, are comforting and help families in the process of healing. Families found memory-making interventions enjoyable and therapeutic, and reported improvements in emotional comfort.^1,16,17,38,39,41,43^ Families described activities involved in memory-making interventions, such as digital storytelling and legacy artwork, as helpful in managing emotions. These practices provided comfort and support during challenging times in palliative care, highlighting the therapeutic value of engaging in memory preservation. This was illustrated by a parent in Akard et al.′s study who stated, *‘I believe it allows families to express their emotions, how treatment from this disease makes them feel. It allows the child to really evaluate how it makes them feel both emotionally and physically’* (^39^, p.7). Parents’ feedback indicated that memory-making interventions like video recordings and heartbeat recordings provided emotional solace to families, offering a structured approach to navigate grief and remember deceased loved ones. Also, memory-making interventions provided emotional relief, helping in grief processing.^1,41,42^

The benefit of memory-making interventions was often gauged through the emotional support they provided and their ability to tailor experiences to individual family needs. Parents valued the therapeutic nature of these interventions, reporting that they assisted with the grieving process and enhanced quality of life by allowing families to maintain a connection with the deceased through personalized legacy items.^16 –18,24,38,39,43^ The transformation of traditional interventions into digital formats extended accessibility and potentially enriched family communication and emotional expression.^39^

##### Enhanced family connection and communication

Seven articles reported on the impact of memory-making interventions on family communication and connection.^21–23,37, 38,39,41^ Families reported that memory-making interventions facilitated open communication, allowing for sharing of experiences that might otherwise remain unspoken. This enhanced communication helped in understanding and supporting each other better through difficult times, as noted in several interventions. For instance, in multiple studies^16–18,39^ many parents acknowledged that the intervention process facilitated communication between them and their child.

Another study found that memory-making interventions not only facilitated family bonding but also opened lines of communication regarding the child’s impending death.^41^ One parent expressed the significance of this experience, stating, ‘*The daddy/mommy/me handprint legacy artwork was very meaningful because it was all of us working on that art as a family. We were creating memories as a family. So that helps to fill a small void in my heart, knowing we have those memories as a family with him*’ (^41^, *p.5*). Memory-making interventions facilitated a sense of closure and enabled families to maintain ongoing bonds with their deceased loved ones, evidenced by tangible memory objects like scrapbooks and legacy videos and strengthened family bonds, providing a shared space for collective grieving and remembrance.

##### Memory preservation and personalization

Six articles highlighted the creation of tangible personalized legacy items such as videos, artwork and digital stories, which were highly valued by families.^24,38–42^ These artefacts served as lasting mementoes that honoured the child’s life and continued to play a role in family dynamics and memory preservation long after the child’s death. Families valued the creation of lasting memories, which were imbued with the child’s personality and the family’s shared experiences.

For example, a parent in Andrews et al.’s study shared, *‘[My daughter] was my centre and we would lay and hold her close to me. The heartbeat recording allows me to live in that memory whenever I want to. Thank you for that endless gift’* (^40^, p.5). Another parent echoed similar sentiments, stating, *‘It keeps her here with me’* (^40^, p.5). These deeply personal stories demonstrate that the legacy items are more than mere keepsakes; they become enduring emotional links to the loved one’s families have lost.

Participants across multiple studies repeatedly emphasized the significance of the meaning behind legacy artwork, which often reflected the child’s personality, cherished memories and the emotions experienced during the creation process.^24,38,39,41,42^ As one parent in Walden et al. ’s study noted, ‘*I have a piece of him that I can hear for forever. It’s not like he’s just gone, and I have to try and imagine it. I can hear it. It’s like a dose of reality; it brings me back to earth, it brings him back to earth for a little bit*’ (^42^, *p.5*). The ability to personalize these interventions ensured that they were closely aligned with the child’s unique identity and the family’s needs, making the experience more meaningful and supportive. This personalization enhanced the overall emotional impact and effectiveness of the interventions.^39^

##### Grieving support and continued bond

Five studies focussing on bereavement care highlighted the role of memory-making interventions in aiding the grief process.^1,24,39,41,42^ Memory-making interventions offered families structured activities that acknowledged their loss and facilitated emotional healing. Through memory-making interventions, families were able to create lasting memories that provided comfort and helped maintain an emotional connection with their deceased loved ones.

One parent in Walden et al.′s study described how these interventions supported their grief journey: *‘Well, I just listen to it because there’s not a day or minute or an hour that goes by where I don’t think about her. I listen to it for comfort, peace and when I go to the cemetery, I play it when I’m standing there looking at her grave’* (^42^, p.6). Another parent similarly stated, *‘It helps me to hold, I mean, that connection is still there’* (^24^, p.6). These examples demonstrate how memory-making interventions help families navigate their grief by preserving a sense of a ‘continued bond’ with the deceased.

For instance, Andrews et al. found that the Music Therapy Heart Sounds programme provided parents with a tangible way to remember and honour their child, described as an ‘*endless gift*’(^40^, *p.5*). that kept their child’s memory alive. As another parent reflected, ‘*The legacy artwork showed that our family is an everlasting circle. Her handprints in the middle of ours represented us surrounding her with love, but at the same time, the butterfly she made with her handprints meant that we were giving her the freedom to fly, take her wings, and go to heaven to be with God. This painting expresses what our family was like in our last moment as one but also tells me that we will all be together again someday*’ (^41^, *p.5).*

Overall, families’ experiences with memory-making interventions highlighted their vital role in providing emotional support, facilitating the grieving process and strengthening family bonds. These interventions are highly valued for their ability to reflect personal and familial identities, adding meaningful support during difficult times.

#### Barriers and enablers affecting implementation

All 11 included articles provided evidence on barriers and enablers to implementing memory-making interventions. Although no studies explicitly focussed solely on these aspects, relevant data were extracted from the results, findings or discussion sections of included papers. Drawing on the template for intervention description and replication checklist, we synthesized barriers and enablers into three main factors related to (i) design and delivery, (ii) participants engagement and (iii) facilitation and support.

##### Design and delivery factors

Eight studies reported that specific design and delivery features of memory-making interventions influenced their implementation and accessibility.^1,16–18,24,39,41 –43^ Some intervention components may have facilitated memory-making interventions’ implementation, including transforming traditional interventions to digital format delivery to offer new avenues for reaching families unable to participate in face-to-face settings, broadening accessibility.^39,43^ On the other hand, technological and accessibility challenges acted as barriers to implementation as digital interventions faced user interface difficulties that negatively impacted user engagement and data collection; and revisions were needed to enhance accessibility and usability of platforms.^24,39^

Design features such as the type of materials used (e.g. heartbeat recordings, handprints or footprints, artwork, photographs, or videos) and the way of delivery (e.g. in person, websites, physical kits specific in hospital) also influenced how well interventions fit within family routines and healthcare workflows.^1,16,18,39^ When the design elements were aligned with the family’s context and the timing of care, implementation was more effective and the interventions were more readily accepted.^1,42^

##### Participants engagement factors

Five studies highlighted the importance of tailoring interventions to individual family preferences, cultural norms and emotional readiness to promote meaningful engagement.^1,17,38,40,42^ When interventions were responsive to children’s personalities and family dynamics, developmental stages and family dynamics they were more likely to be perceived as meaningful and supportive. For example, one study noted how families valued personalized artefacts that reflected their child’s identity.^1^ Another reported that heartbeat recordings became cherished, ongoing sources of connection.^42^ Personalization, particularly when aligned with family time and values (e.g. desire for earlier intervention or proximity to the child),^40^ cultural background (e.g. parental interpretations of heartbeat recordings as spiritual or sacred experiences)^42^ and the child’s unique traits or developmental stage was consistently reported as a key factor in enhancing participation and acceptability.^38^ Involving families in shaping the intervention and ensuring cultural and personal relevance further strengthened engagement and satisfaction.^1,42^

##### Facilitation and support factors

Three studies highlighted the importance of facilitation and support; particularly emotional support in enabling successful implementation of memory-making interventions.^24,41,42^ These factors include the emotional readiness of parents, the supportive nature of the care environment and active facilitation by healthcare professionals.

In several cases, memory-making activities were supported by trained facilitators (e.g. art or music therapists), whose expertise and sensitivity were important in guiding families through the emotionally intense process of legacy creation. The involvement of trained facilitators helped the interventions to be both meaningful and manageable for families.^41,42^

Engaging in legacy-making can be deeply impactful and, at times, overwhelming. For some parents, this emotional intensity limited their ability to participate consistently.^41^ However, when accompanied by appropriate emotional support, these interventions offered significant comfort and contributed to the grieving and healing process.^24^ Strategies such as digital storytelling and therapeutic videography were found to facilitate coping and emotional expression. A supportive and private environment further enhanced the intervention’s impact.^41^

## Discussion

### Summary of findings

This review synthesized qualitative and quantitative evidence on the use of memory-making interventions for children with life-limiting or life-threatening conditions and their families, in paediatric palliative and bereavement care, examining their implementation, family experiences and reported outcomes. Interventions such as digital storytelling, art therapy, videography and the creation of physical keepsakes have been explored as ways to help meet the complex emotional and psychological needs of families navigating paediatric palliative and bereavement care. These memory-making interventions were found to have potential positive effects on emotional coping, family communication and memory preservation, highlighting their importance as psychosocial support tools during these critical periods.

Memory-making interventions can serve as therapeutic tools that facilitate emotional expression while creating valuable artefacts that families can hold onto, providing comfort and maintaining a connection with their deceased child.^27^ This dual function—supporting coping during illness and remembrance after death—is especially relevant in paediatric palliative settings, where families often experience anticipatory grief alongside caregiving demands. While not all studies focussed explicitly on anticipatory grief, several offered indirect insights. For example, some studies reported that digital storytelling supported children in expressing emotions and sharing their identities while living with life-limiting or life-threatening conditions in palliative care settings.^16,17,38^ Other studies highlighted how memory-making activities near the end of life offered emotional preparation for loss and described how heartbeat recordings supported a continued sense of connection during the progression of neurodegenerative conditions.^42^ Art-based interventions, such as memory boxes, handprint art and personalized jewellery, were especially valued by families for their emotional significance and symbolic role in honouring the child’s identity and preserving shared memories, which families found deeply meaningful both during the child’s illness and in bereavement care.^1^ These findings reflect the principles of continuing bonds theory,^44^ which emphasizes the importance of maintaining emotional ties with the deceased as part of healthy grief processing.

Qualitative feedback from participating families emphasized the importance of personalized and culturally sensitive approaches in memory-making interventions. Families emphasized that the effectiveness of memory-making interventions is closely linked to how well they are tailored to meet individual preferences and cultural needs. For instance, families who engaged in videography projects valued the opportunity to capture and preserve their child’s voice and personality, which provided significant comfort and a sense of continuity during the bereavement process.^45,46^ This personalized nature of memory-making aligns with findings from Xu et al. who highlighted the significance of these practices in connecting families with their children and navigating grief.^30^

### What this study adds

This review builds on existing literature by highlighting the critical importance of tailored approaches that consider the specific needs and preferences of families. While prior research has demonstrated the general benefits of memory-making activities, this review adds to the understanding by identifying the key factors that enhance or hinder their effectiveness. Importantly, it also shows that memory-making interventions serve overlapping but distinct roles across care phases, supporting anticipatory grief and communication during palliative care,^1^ and fostering continued bonds in bereavement care.^24,41^ The findings suggested that successful implementation of memory-making interventions is dependent on the level of personalization, cultural sensitivity and the provision of adequate support. This focus on customization is an important contribution, as it stresses the need for healthcare providers to be flexible and responsive to the unique circumstances of each family.

Despite the generally positive experiences reported by families, this review also identified several barriers to the successful implementation of memory-making interventions. These barriers include technological challenges, such as accessibility issues and user interface problems, as well as cultural sensitivities and personal preferences that can impact the uptake and effectiveness of these interventions.^39,43^ Some families felt unprepared for memory-making or wished it had started earlier.^1,42^ We recommend offering interventions at flexible times and involving families in planning, so their needs and preferences are better met. Xu et al. similarly found that a lack of understanding and preparation could prevent families from fully benefitting from these interventions, emphasizing the need for improved support and culturally informed practices.^30^

Conversely, the review highlighted several enablers of successful implementation. These include the involvement of trained facilitators, the provision of emotional and technical support and the creation of supportive and private environments for families.^40,43^ The active involvement of healthcare providers, particularly their sensitivity in guiding families through the memory-making process, is crucial for the success of these interventions. Moreover, findings from some studies indicate that broader inclusion criteria ^17,18,39^; such as allowing participation from children at various stages of illness or from broader demographic groups alongside personalized approaches, can enhance engagement, making memory-making interventions more accessible and meaningful for a diverse range of families.

### Strengths and limitations

This review’s strength lies in its synthesis of evidence on memory-making interventions in paediatric palliative and bereavement care, addressing a notable gap with a focus on adaptability to family needs and cultural contexts. Healthcare providers need to adopt a flexible approach, tailoring interventions to individual preferences to maximize psychosocial benefits, such as family cohesion, emotional regulation and continued bonds during bereavement. Based on our findings, we recommend that clinical teams offer families a range of memory-making options (e.g. heartbeat recordings, digital stories, or handprint art), initiate these conversations early in the care journey, provide emotional and technical support, and adapt interventions to cultural preferences and developmental stages. Supporting this approach will also require healthcare staff training in grief communication and culturally responsive psychosocial care. The review also called for further research to develop culturally sensitive and developmentally appropriate interventions to better support families.

There are several limitations to consider. Although seven included articles^16 –18,24,38,39,43^ used quantitative methods, and five of them were randomized controlled trials, none demonstrated statistically significant effects. This limits confidence in the findings and highlights the need for further experimental research to determine the efficacy of these interventions as psychosocial support tools during these critical periods.

While some studies included children and young people as participants,^16 –18,38,39,43^ their involvement was typically limited to structured quantitative measures. No included studies provided qualitative data that captured children’s voices directly through narrative or quotations. This lack of qualitative insight from children limits our understanding of their subjective experiences with memory-making interventions and highlights a significant gap in the evidence base. Future research should prioritize the inclusion of child and young people perspectives through appropriate and ethically sound qualitative methods.

Another limitation is the relatively small number of studies that focussed primarily on parents as participants,^1,24,41,42^ particularly in the context of bereavement care. While some studies did include bereaved parents,^1,24,41,42^ this area remains underrepresented. This limited parental focus restricts our understanding of how memory-making interventions impact family dynamics and the grieving process, suggesting a need for more parent-centred research in future studies. Additionally, the exclusion of non-English language studies may have led to a publication bias.

## Conclusion

Memory-making interventions are a crucial component of holistic paediatric palliative and bereavement care. Although this review found limited statistically significant results, qualitative data suggested that these interventions provide numerous positive benefits, including emotional and psychosocial support. Memory-making activities have been described by families as supportive in documenting and preserving meaningful memories, potentially aiding in grief coping and fostering resilience. The review emphasized the need for personalized and culturally sensitive approaches and calls for further research to address implementation challenges and establish standardized practices. Optimizing memory-making interventions has the potential to meaningfully improve the quality of life for children with life-limiting or life-threatening conditions and their families, ensuring comprehensive support throughout their palliative and bereavement journeys.

## Supplemental Material

sj-docx-1-pmj-10.1177_02692163251353006 – Supplemental material for Memory-making interventions for children with life-threatening or life-limiting conditions and their families: A systematic review of evidence and implications for practice Supplemental material, sj-docx-1-pmj-10.1177_02692163251353006 for Memory-making interventions for children with life-threatening or life-limiting conditions and their families: A systematic review of evidence and implications for practice by Razieh Safarifard, Gemma Kiernan, Yvonne Corcoran, Eileen Courtney, John Mitchell, Terrah Akard and Veronica Lambert in Palliative Medicine
