# Supplementary material for: Memory-making interventions for children with life-threatening or life-limiting conditions and their families: A systematic review of evidence and implications for practice
Source: Palliat Med. 2025 Jul 25;39(8):871–83. doi: 10.1177/02692163251353006 (PMC12405643; doi:10.1177/02692163251353006)
Supplement: sj-docx-1-pmj-10.1177_02692163251353006 – Supplemental material for Memory-making interventions for children with life-threatening or life-limiting conditions and their families: A systematic review of evidence and implications for practice [file sj-docx-1-pmj-10.1177_02692163251353006.docx]

**Memory-making interventions for children, young people and their families receiving paediatric palliative or bereavement care: a systematic review**

**Description of Supplemental Files**

**Supplemental File 1.** Sample Search Strategy and the search strategy used in Scopus

**Supplemental File 2.** PRISMA 2020 checklist

**Supplemental File 3.** Summary of included studies

**Supplemental File 4.** The Template for Intervention Description and Replication (TIDieR) checklist

**Supplemental File 5.** Details of the Mixed Methods Appraisal Tool (MMAT) evaluations

**Supplemental File1.** Sample Search Strategy

| **Search ID** | **Search Terms** |
| --- | --- |
| S1 | legacy OR "legacy making" OR "legacy-making" OR "memory making" OR "memory-making" OR "legacy building*" OR "legacy activit*" OR "dignity therap*" OR "legacy service*" OR "life review" |
| S2 | child* OR teen* OR youth* OR adolescent* OR "under 19" OR "under 18" OR infant* OR newborn* OR "new born*" OR "new-born*" |
| S3 | pediatric* OR paediatric* OR parent* OR sibling* OR famil* OR "family members" |
| S4 | S2 OR S3 |
| S5 | intervention* OR therap* OR treatment* OR "activit*" |
| S6 | bereavement OR bereave* OR grief OR mourning OR "grief support" OR "mourning support |
| S7 | S5 OR S6 |
| S8 | S1 AND S4 AND S7 |

An example of a search term used in Scopus was:

(TITLE-ABS-KEY (legacy OR "legacy making" OR "legacy-making" OR "memory making" OR "memory-making" OR "legacy building*" OR "legacy activit*" OR "dignity therap*" OR "legacy service*" OR "life review")) AND (TITLE-ABS-KEY (parent* OR sibling* OR famil* OR "family members" OR child* OR teen* OR youth* OR adolescent* OR "under 19" OR "under 18" OR infant* OR newborn* OR "new born*" OR "new-born*" OR pediatric* OR paediatric*)) AND (TITLE-ABS-KEY (intervention* OR therap* OR treatment* OR "activit*" OR bereavement OR bereave* OR grief OR mourning OR "grief support" OR "mourning support")).

**Supplemental File 2.** PRISMA 2020 checklist

| **Section and Topic** | **Item #** | **Checklist item** | **Location where item is reported** |
| --- | --- | --- | --- |
| **TITLE** | | |  |
| Title | 1 | Identify the report as a systematic review. | 1 |
| **ABSTRACT** | | |  |
| Abstract | 2 | See the PRISMA 2020 for Abstracts checklist. | 2 |
| **INTRODUCTION** | | |  |
| Rationale | 3 | Describe the rationale for the review in the context of existing knowledge. | 4-5 |
| Objectives | 4 | Provide an explicit statement of the objective(s) or question(s) the review addresses. | 5 |
| **METHODS** | | |  |
| Eligibility criteria | 5 | Specify the inclusion and exclusion criteria for the review and how studies were grouped for the syntheses. | 6 |
| Information sources | 6 | Specify all databases, registers, websites, organisations, reference lists and other sources searched or consulted to identify studies. Specify the date when each source was last searched or consulted. | 5 |
| Search strategy | 7 | Present the full search strategies for all databases, registers and websites, including any filters and limits used. | 5+Supplemental files |
| Selection process | 8 | Specify the methods used to decide whether a study met the inclusion criteria of the review, including how many reviewers screened each record and each report retrieved, whether they worked independently, and if applicable, details of automation tools used in the process. | 5-7 |
| Data collection process | 9 | Specify the methods used to collect data from reports, including how many reviewers collected data from each report, whether they worked independently, any processes for obtaining or confirming data from study investigators, and if applicable, details of automation tools used in the process. | 5-7 |
| Data items | 10a | List and define all outcomes for which data were sought. Specify whether all results that were compatible with each outcome domain in each study were sought (e.g. for all measures, time points, analyses), and if not, the methods used to decide which results to collect. | 9-10+Supplemental files |
|  | 10b | List and define all other variables for which data were sought (e.g. participant and intervention characteristics, funding sources). Describe any assumptions made about any missing or unclear information. | 9-10 |
| Study risk of bias assessment | 11 | Specify the methods used to assess risk of bias in the included studies, including details of the tool(s) used, how many reviewers assessed each study and whether they worked independently, and if applicable, details of automation tools used in the process. | 11 |
| Effect measures | 12 | Specify for each outcome the effect measure(s) (e.g. risk ratio, mean difference) used in the synthesis or presentation of results. | - |
| Synthesis methods | 13a | Describe the processes used to decide which studies were eligible for each synthesis (e.g. tabulating the study intervention characteristics and comparing against the planned groups for each synthesis (item #5)). | 8-10 |
|  | 13b | Describe any methods required to prepare the data for presentation or synthesis, such as handling of missing summary statistics, or data conversions. | 7 |
|  | 13c | Describe any methods used to tabulate or visually display results of individual studies and syntheses. | - |
|  | 13d | Describe any methods used to synthesize results and provide a rationale for the choice(s). If meta-analysis was performed, describe the model(s), method(s) to identify the presence and extent of statistical heterogeneity, and software package(s) used. | 7 |
|  | 13e | Describe any methods used to explore possible causes of heterogeneity among study results (e.g. subgroup analysis, meta-regression). | - |
|  | 13f | Describe any sensitivity analyses conducted to assess robustness of the synthesized results. | - |
| Reporting bias assessment | 14 | Describe any methods used to assess risk of bias due to missing results in a synthesis (arising from reporting biases). | 7-9 |
| Certainty assessment | 15 | Describe any methods used to assess certainty (or confidence) in the body of evidence for an outcome. | 7-9 |
| **RESULTS** | | |  |
| Study selection | 16a | Describe the results of the search and selection process, from the number of records identified in the search to the number of studies included in the review, ideally using a flow diagram. | 9 |
|  | 16b | Cite studies that might appear to meet the inclusion criteria, but which were excluded, and explain why they were excluded. | 9 |
| Study characteristics | 17 | Cite each included study and present its characteristics. | 9-10 |
| Risk of bias in studies | 18 | Present assessments of risk of bias for each included study. | 11 |
| Results of individual studies | 19 | For all outcomes, present, for each study: (a) summary statistics for each group (where appropriate) and (b) an effect estimate and its precision (e.g. confidence/credible interval), ideally using structured tables or plots. | 9-10 |
| Results of syntheses | 20a | For each synthesis, briefly summarise the characteristics and risk of bias among contributing studies. | 9-11 |
|  | 20b | Present results of all statistical syntheses conducted. If meta-analysis was done, present for each the summary estimate and its precision (e.g. confidence/credible interval) and measures of statistical heterogeneity. If comparing groups, describe the direction of the effect. | 9-11 |
|  | 20c | Present results of all investigations of possible causes of heterogeneity among study results. | 9-10 |
|  | 20d | Present results of all sensitivity analyses conducted to assess the robustness of the synthesized results. | - |
| Reporting biases | 21 | Present assessments of risk of bias due to missing results (arising from reporting biases) for each synthesis assessed. | 11+ Supplemental files |
| Certainty of evidence | 22 | Present assessments of certainty (or confidence) in the body of evidence for each outcome assessed. | 9-10+Supplemental files |
| **DISCUSSION** | | |  |
| Discussion | 23a | Provide a general interpretation of the results in the context of other evidence. | 16 |
|  | 23b | Discuss any limitations of the evidence included in the review. | 16-17 |
|  | 23c | Discuss any limitations of the review processes used. | 16-17 |
|  | 23d | Discuss implications of the results for practice, policy, and future research. | 17 |
| **OTHER INFORMATION** | | |  |
| Registration and protocol | 24a | Provide registration information for the review, including register name and registration number, or state that the review was not registered. | 5 |
|  | 24b | Indicate where the review protocol can be accessed, or state that a protocol was not prepared. | - |
|  | 24c | Describe and explain any amendments to information provided at registration or in the protocol. | 5 |
| Support | 25 | Describe sources of financial or non-financial support for the review, and the role of the funders or sponsors in the review. | 17 |
| Competing interests | 26 | Declare any competing interests of review authors. | 17 |
| Availability of data, code and other materials | 27 | Report which of the following are publicly available and where they can be found: template data collection forms; data extracted from included studies; data used for all analyses; analytic code; any other materials used in the review. | 9-10- supplemental file |

**Supplemental File 3.** Summary of included studies

| **ID** | **Author (Year)** | **Year** | **Country** | **Aim** | **Design** | **Participant Demographics/ Participant Characteristics** | **Age (Mean, Range)** | **Place of Intervention** | **Setting** | **Context** | **Interventions** | **Comparators** | **Outcome Measures** | **Main outcomes** | **MMAT Score** |
| --- | --- | --- | --- | --- | --- | --- | --- | --- | --- | --- | --- | --- | --- | --- | --- |
| **1** | Akard et al.^38^ (2015) | 2015 | USA | Assess a legacy-making intervention's impact of Quality of life on children with cancer | Quantitative RCT | 28 Children with cancer, ages 7–17, and their parents | 11, 7-17 | Home | At the child’s home or another chosen private location | Pediatric palliative Care | Digital storytelling intervention focusing on legacy-making | Usual care | Quality of life- PedsQL for QoL, Parent and child interviews for experiences and perceptions | Feasibility of digital storytelling; preliminary trends toward improved emotional and school functioning and quality of life (not statistically significant)​ | High (5/5) |
| **2** | Akard et al.^24^ (2018) | 2018 | USA | Explore bereaved parents’ perceptions of a digital storytelling legacy-making intervention | Qualitative -No specific design stated | 6 bereaved parents (3 mothers and 3 fathers) of infants who received care or died in the NICU | 29.5(Parents) | NICU | The NICU setting or at any chosen location by the bereaved family for interviews or capturing moments | Bereavement Care | Digital storytelling legacy-making intervention for parents after the death of an infant | N/A | Parents' perceptions and suggestions for the legacy intervention; feasibility, acceptability, and potential benefits | Parents reported digital storytelling as acceptable and beneficial for grief processing | High (5/5) |
| **3** | Akard et al.^39^(2020) | 2020 | USA | Describe the development of a web-based legacy intervention for children with relapsed/refractory cancer | Quantitative RCT (Feasibility) | 81 children aged 7-17 with relapsed/refractory cancer and their primary parent caregivers | 10, 7-17 (children) | Web-based platform | Accessible from any location with internet access, enabling participation from home or any preferred location. | Pediatric palliative Care | Web-based program guiding children to create digital stories about themselves | Usual care | Feasibility, acceptability, parent reports on satisfaction surveys, and suggestions for future improvements | High engagement and feasibility of web-based intervention | High (5/5) |
| **4** | Andrews et al.^40^(2020) | 2020 | USA | Examine the impact of the Music Therapy Heart Sounds (MTHS) program on bereaved parents | Quantitative non-randomized | 12 bereaved parents of children treated at a tertiary care children's hospital; most respondents (10) were mothers | 7 (children) | children’s Hospital | The intervention took place at a tertiary care children’s hospital. | Bereavement Care | The Music Therapy Heart Sounds (MTHS) program recording children’s heartbeats using a digital stethoscope and creating mementos | N/A | Five-question survey assessing parents' awareness of the program, usage of the heartbeat recordings, and the program's impact on their grief process. | Memory-making supported grieving and memory preservation | High (4/5) |
| **5** | Schaefer et al.^41^(2020) | 2020 | USA | Explore legacy-making and grief experiences of bereaved parents who participated in legacy artwork | Qualitative- Conventional content analysis | 12 bereaved parents (mothers comprising 83%) and 12 healthcare providers from a children's hospital | 41.17 (parents) | children’s Hospital | The intervention took place at a pediatric oncology hospital, with interviews likely conducted in settings convenient for participants. | Bereavement care/Subjects: Tertiary care children's hospital | Legacy artwork interventions provided by the hospital's interdisciplinary pediatric oncology team. | N/A | Semi-structured individual interviews analyzed via conventional content analysis. Parental experiences and perceptions of the legacy artwork's impact on their grief processes. | Legacy art provided emotional healing and enduring connection with deceased children | High (5/5) |
| **6** | Akard et al.^16^(2021) | 2021 | USA | To examine the impact of a web-based legacy intervention on parent–child communication among children with relapsed or refractory cancer. | Quantitative RCT | 150 Randomized Children aged 7–17 with relapsed or refractory cancer and their primary parent caregivers. Majority were female (59%) and Caucasian (85%). | 10, 7-17 (children) | Web-based platform | Accessible from any location with internet access, enabling participation from home or any preferred location. | Pediatric palliative Care | Web-based program guiding children to create digital stories about themselves | Usual care | Parent–Adolescent Communication Scale (PACS) assessing open communication, problems in communication, and overall communication between parent and child. | Improved parent–child communication, especially with fathers (not statistically significant) | High (5/5) |
| **7** | Akard et al.^17^(2021) | 2021 | USA | To examine the effects of a legacy intervention for children with advanced cancer and their parents on parental coping strategies. | Quantitative RCT | 150 children with advanced cancer and their parents, recruited via Facebook. Children aged 7–17 years. | 10, 7-17 (children) | Web-based platform | Accessible from any location with internet access, enabling participation from home or any preferred location. | Palliative Care for families | Web-based legacy intervention where children create digital storyboards documenting their legacies | Usual care. | Parental coping strategies measured by the Responses to Stress Questionnaire at baseline/pre intervention (T1) and post intervention (T2). | Improved parental coping, small trends toward increased primary control and disengagement coping (not statistically significant) | High (5/5) |
| **8** | Akard et al.^18^(2021) | 2021 | USA | Examine the impact of a web-based legacy intervention on Quality of Life (QOL) in children with advanced cancer | Quantitative RCT | 150 child-parent dyads recruited through Facebook, with 97 dyads included in the analysis | 10, 7-17 (children) | Web-based platform | Accessible from any location with internet access, enabling participation from home or any preferred location. | Pediatric palliative Care | Web-based legacy-making intervention guiding children to create digital storyboards about themselves. | Usual care | Quality of Life (QOL) in children with advanced cancer, utilizing the PedsQL Cancer Module pre and post-intervention, including various QOL dimensions |  | High (5/5) |
| **9** | Clarke and Connolly.^1^(2021) | 2022 | Ireland | Explore the lived experience of a memory-making process on parents of children at or near end-of-life | Qualitative- Interpretative Phenomenological Analysis | 6 parents (all mothers) whose child had died and who had engaged in memory making, drawn from a children’s hospice. | 3 days-10 years (children) | Children’s Hospice. | Sessions took place primarily in the child’s home or at the hospital, providing a comfortable and private environment for the families. | Bereavement Care | Memory-making interventions, including creating individual pieces of art capturing participation of family members. | N/A | Individual interviews conducted; thematic analysis of interview transcripts exploring parents' experiences with memory-making activities. |  | High (5/5) |
| **10** | Walden et al.^42^(2021) | 2021 | USA | Explore the lived experience of Heartbeat Recordings (HBRs) for bereavement in the lives of parents of children | Qualitative- Thematic analysis informed by mind mapping | 11 English-speaking parents of children with PNDI receiving palliative care services. | 20-68 (parents) | Academic Pediatric Hospital. | The intervention took place within a pediatric hospital setting, specifically within the palliative care department. | Bereavement Care | Heartbeat recordings synchronized with a favorite song, added to the patient's or parent's favorite music. | N/A | Semi-structured interviews conducted three months after receiving their child’s HBR; data collection concurrent with the mind mapping process. |  | High (4/5) |
| **11** | Cho et al.^43^(2023) | 2023 | USA | Assess a digital storytelling-legacy intervention's impact on children's coping | Quantitative RCT | 150 children with recurrent or refractory cancer and their parents, recruited via Facebook. Children aged 7–17 years. | 10, 7-17 (children) | Web-based platform | The intervention was web-based, allowing participation from any location with internet access. | Pediatric palliative Care | Digital storytelling-legacy intervention vs. waitlist control | Usual care | Adaptive coping measured by changes in primary-control and disengagement coping strategies among children, Response to Stress Questionnaire (RSQ) |  | High (5/5) |

**Supplemental File 4.** The Template for Intervention Description and Replication (TIDieR) checklist

| **TIDieR Item** | **Description** | **Akard et al.^37^** | **Akard et al.^13^** | **Akard et al. ^38^** | **Akard et al. ^21^** | **Akard et al. ^22^** | **Akard et al. ^23^** | **Cho, et al. ^39^** | **Andrews et al. ^40^** | **Schaefer et al.^42^** | **Clarke and Connolly.^18^** | **Walden et al.^41^** |
| --- | --- | --- | --- | --- | --- | --- | --- | --- | --- | --- | --- | --- |
| **BRIEF NAME** | Provide the name or a phrase that describes the intervention. | Digital storytelling legacy-making intervention | Digital storytelling legacy-making intervention for parents after the death of an infant in the NICU | Web-Based Legacy Intervention for Children with Advanced Cancer | Web-based legacy intervention for children with advanced cancer | Web-Based Pediatric Oncology Legacy Intervention | Web-Based Legacy Intervention for Children with Advanced Cancer | Web-Based Pediatric Oncology Legacy Intervention | Music Therapy Heart Sounds (MTHS) Program | Legacy Artwork in Pediatric Oncology | Memory Making at End of Life | Heartbeat Recordings (HBR) in Music Therapy |
| **WHY** | Describe any rationale, theory, or goal of the elements essential to the intervention. | Improve quality of life (QOL) for children with cancer by allowing them to create digital stories as a form of legacy-making. | To offer bereaved NICU parents a way to memorialize their infants, support their grieving process, and potentially improve coping mechanisms through legacy-making. | To examine the feasibility and acceptability of a web-based legacy intervention aimed at reducing suffering and distress among seriously ill children and their families by enabling them to create digital stories. | To improve parent–child communication following the diagnosis of advanced cancer in children. Legacy-building as a priority for quality palliative care. | This intervention aimed to help parents cope with the emotional distress associated with their child's advanced cancer through legacy-making activities. | The intervention aimed to improve quality of life (QOL) for children with advanced cancer through legacy-making activities that include digital storytelling. | To explore the effects of a digital storytelling-legacy intervention on adaptive coping strategies in children with recurrent or refractory cancer. | To explore the impact of a legacy-making intervention on bereaved parents by using a digital stethoscope to record the heartbeats of children approaching end-of-life. | To explore the impact of creating legacy artwork on the grief experiences of bereaved parents, aiming to enhance communication, meaning-making, and emotional healing. | To explore parents' lived experiences of memory-making with their child at or near the end of life and the impact of these activities on their bereavement process. | To explore the impact of heartbeat recordings on the bereavement process of parents whose children suffer from progressive neurodegenerative illnesses. |
| **WHAT (Materials)** | Describe any physical or informational materials used in the intervention, including those provided to participants or used in intervention delivery or in training of intervention providers. | Video recordings, music, and photographs chosen by the child. | Digital storytelling tools, including video recordings of the infant and interviews with the parents. | Web-based program for creating digital stories, including components for children to answer legacy questions about themselves, upload photographs and videos, and select music for their digital story. | Web-based platform allowing children to create digital storyboards by answering legacy questions about themselves, uploading photographs, videos, and music. | Digital storytelling tools including electronic digital storyboards that allowed for uploading of videos, pictures, and text. | Digital tools were used to create storyboards that included personal narratives, photographs, videos, and music uploaded by the children. | Digital storytelling tools enabling the creation of personal storyboards with uploads of photographs, videos, and text. | The Eko CORE digital stethoscope was used to record heartbeats, which were then integrated into personalized mementos such as songs, voice recordings, or standalone phonocardiograms. | Art supplies for creating physical artwork, which included paintings and possibly other mediums, personalized by the children and their families. | Memory-making materials used included items for creating tangible mementos such as hand and footprints, locks of hair, and other personalized items that capture aspects of the child's presence. | Digital stethoscope used to record the child’s heartbeat, which was then synchronized with a favorite song selected by the parents. |
| **WHAT (Procedures)** | Describe each of the procedures, activities, and/or processes used in the intervention, including any enabling or support activities. | Videographers record children's responses to interview questions about their legacy, incorporating their chosen music and photographs to create a digital story. | Parents were introduced to the idea of digital storytelling through examples. Suggested procedures for creating digital stories for their infants were discussed. Focus group interviews with bereaved parents to gather insights on the development, feasibility, and acceptability of the proposed intervention. | Children and their parents were guided to create a digital story through a web-based platform. This process included selecting and answering guiding legacy questions, uploading personal photographs and videos, and selecting background music to compile into a digital story. | The intervention website guided children to create digital storyboards over 2 weeks, directing them to answer legacy questions and upload multimedia content. Families received a copy of the child's final digital story. | Parents and children created digital stories that documented the child's legacy through guided questions, personal traits, likes, connections, uploading personal photographs and videos, and selecting music. | Children were guided to create digital storyboards about themselves, documenting personal characteristics, activities they like, and connections with others. The intervention was designed to be completed within two weeks. | Participants developed their digital stories by responding to prompts about their personal characteristics, interests, and relationships. They could upload relevant multimedia to enhance their stories, which were then formatted cinematically on the web platform. | Music therapists recorded children's heartbeats, edited ambient noise to isolate the heartbeat, and overlaid it with a chosen song or voice recording. Art therapists created artistic embellishments of the phonocardiogram. | Bereaved parents and healthcare providers participated in interviews to discuss their experiences with the legacy artwork created before the child's death. The intervention involved creating artwork that reflected the child's personality and preferences. | Parents were introduced to memory-making activities by healthcare professionals who facilitated the creation of various keepsakes. These sessions were conducted either at the child's home or in the hospital depending on the child's condition. | Music therapists recorded the child's heartbeat using a digital stethoscope, consulted with parents to choose a significant song, and combined the heartbeat with the music to create a personalized recording. |
| **WHO PROVIDED** | For each category of intervention provider (e.g., psychologist, nursing assistant), describe their expertise, background, and any specific training given. | Conducted by researchers and a videographer, developed based on suggestions from children with cancer and their parents. | The study was conducted by a research team from Vanderbilt University, including experts in nursing, bereavement, and pediatric palliative care. | The intervention was developed and overseen by a team including an associate professor of nursing and medicine with contributions from experts in pediatric oncology, biostatistics, and palliative care nursing. | The study was conducted by a team from Vanderbilt University School of Nursing, Medicine, and Medical Center, The Ohio State University, Michigan State University, Saint Louis University, Children's National Health System, and The George Washington University. | The study was conducted by a multidisciplinary research team from Vanderbilt University, with experts in nursing, pediatric oncology, biostatistics, and palliative care. | The study was conducted by a multidisciplinary team from Vanderbilt University, including experts in nursing, biostatistics, pediatric oncology, and palliative care. | The intervention was administered by a research team including experts in nursing, pediatric oncology, biostatistics, and palliative care from Vanderbilt University and other collaborating institutions. | The intervention was provided by a multidisciplinary team including pediatric palliative care physicians, music therapists, and child life specialists. | The study was facilitated by healthcare providers including art therapists at a pediatric hospital. They guided the creation of legacy artwork. | The memory-making sessions were provided by a team comprising nursing staff and other healthcare professionals trained in end-of-life care and family support. | Music therapists at an academic pediatric hospital provided the intervention, with support from the palliative care team. |
| **HOW** | Describe the modes of delivery (e.g., face-to-face or by some other mechanism such as internet or telephone) of the intervention and whether it was provided individually or in a group. | Individual sessions at the patient’s home or another private location, utilizing digital means for story creation and sharing. | The intervention seems designed for individual families. The intervention concept was presented through focus group discussions, with digital storytelling examples shown to participants. | The intervention was delivered entirely online through a specially designed web-based platform accessible to the participating children and their parents. | Delivered entirely online through a web-based platform, accessible to participating children and their parents. | The intervention was delivered via a web-based platform, with interactions primarily occurring online. | Delivered entirely online through a secure web-based platform that participants could access to input their stories and media. | The intervention was delivered online, with participants accessing the intervention website to create and submit their digital stories. | The intervention involved recording the heartbeat using a digital stethoscope, editing it, and presenting it to parents in various formats. | Interviews were conducted face-to-face or via telephone to gather data on the impact of the legacy artwork. | The activities were conducted face-to-face, allowing parents to actively participate in creating mementos of their child. | The intervention was delivered through a combination of music therapy techniques, involving the recording of heartbeats and music mixing. |
| **WHERE** | Describe the type(s) of location(s) where the intervention occurred, including any necessary infrastructure or relevant features. | At the child’s home or another chosen private location. | The intervention, as discussed, would take place within the NICU setting or any chosen location by the bereaved family for interviews or capturing moments. Specifics were not provided. | The intervention was accessible from any location with internet access, allowing participants to engage with the intervention from home or any preferred location. | Accessible from any location with internet access, enabling participation from home or any preferred location. | Accessible from any location with internet access, allowing participants to engage with the intervention remotely. | The intervention was accessible from any location with internet access, facilitating participation from home or any location convenient to the participants. | The intervention was web-based, allowing participation from any location with internet access. | The intervention took place at a tertiary care children’s hospital. | The intervention took place at a pediatric oncology hospital, with interviews likely conducted in settings convenient for participants. | The sessions took place primarily in the child’s home or at the hospital, providing a comfortable and private environment for the families. | The intervention took place within a pediatric hospital setting, specifically within the palliative care department. |
| **WHEN and HOW MUCH** | Describe the number of times the intervention was delivered and over what period of time, including the number of sessions, their schedule, and their duration, intensity, or dose. | Single session intervention, with editing time around 6 hours per story; final digital stories averaged 7 minutes 40 seconds. | The concept involves creating digital stories during the NICU stay or shortly after an infant's death. | The intervention was designed to be completed at the participants' own pace, with children asked to complete their digital story within two weeks of starting. | Designed to be completed at the participants' own pace within two weeks of starting. | The intervention was intended to be completed within two weeks of initiation, with children and parents creating the digital story collaboratively. | The intervention was intended to be brief, with participants asked to complete their digital stories within a two-week period after initiation. | The intervention was designed to be completed within two weeks, with flexibility allowed for the complex nature of the participants' medical conditions. | Parents participated in a single session to create the heartbeat recording, and additional follow-up was available for creating original music or personal artwork. | The legacy artwork was created at various times before the child's passing, as remembered and reported by the participants during interviews. | The memory-making activities occurred during the final days of the child’s life or shortly after, usually over several sessions depending on the family's needs and the child’s health condition. | Parents received their child’s heartbeat recording three months prior to the interviews; the creation process timing was based on the child’s health status. |
| **TAILORING** | If the intervention was planned to be personalized, titrated, or adapted, then describe what, why, when, and how. | Highly personalized, tailored to each child’s preferences for music, photographs, and content shared in their digital story. | Suggested to be highly personalized, allowing parents to share their infant's story and incorporate personal memories and messages, but specifics are not detailed. | The intervention was highly personalized, allowing each child to choose the content, photographs, videos, and music that best represented their personal story and legacy. | Highly personalized, allowing each child to choose the content, photographs, videos, and music for their digital story. | Highly personalized, allowing for the inclusion of content specifically chosen by the children and their parents to reflect personal memories and messages. | Highly personalized, as the intervention allowed children to choose the content, pictures, videos, and music that they felt best represented their legacy. | Highly personalized, with children choosing the content of their stories based on personal preferences without focus on their medical condition or treatment. | The intervention was highly personalized, allowing families to choose songs, contribute to the artwork, and decide how the heartbeat recording would be used. | Highly personalized; the artwork was tailored to each child’s interests and characteristics, often involving input from the child when possible. | Each session was highly personalized, focusing on the preferences and emotional comfort of the family and the child. | Highly personalized, with each recording tailored to include the child's heartbeat synchronized with a song that held personal significance to the family. |
| **MODIFICATIONS** | If the intervention was modified during the course of the study, describe the changes (what, why, when, and how). | Possible minor edits to digital stories based on family feedback; one family requested edits to include more family pictures and an "in memory" page. | Suggestions for intervention development were based on parents’ feedback, focusing on feasibility, acceptability, and personalized content. | Modifications to the web platform were made to improve user-friendliness and address technical issues. Additional enhancements included the development of a cinematic feature for the digital stories. | Adjustments made to the web platform to improve user-friendliness and address technical issues based on participant feedback. | Adjustments made to the web-based platform to improve user-friendliness based on participant feedback. | Not detailed in the document, but typical web-based interventions may involve adjustments based on user feedback to improve accessibility and engagement. | Not specifically mentioned in the document. | No modifications to the intervention process were reported during the study. | Not explicitly detailed; however, the personalized nature of the artwork implies adaptability based on the child’s and family's preferences and needs. | Not specified in the details provided; however, the personalized nature of the sessions suggests that adjustments were likely made based on the family's requests and the child’s condition. | Not specified, but the intervention was likely adapted to each child's condition and family preferences during the song selection process. |
| **HOW WELL (Planned)** | If intervention adherence or fidelity was assessed, describe how and by whom, and if any strategies were used to maintain or improve fidelity, describe them. | Intervention fidelity maintained through direct delivery or review of unedited videos by the PI, and following a delivery guide. | The study did not detail plans for assessing adherence or fidelity to the proposed intervention design. The concept and structure of the intervention were developed based on qualitative feedback from bereaved parents, aiming for high relevance and sensitivity. | The intervention's delivery was closely monitored for adherence to the planned procedures and modifications were made based on participant feedback and technical requirements. | Monitored for adherence to planned procedures; modifications were made to enhance the intervention based on technical requirements and participant feedback. | The intervention's delivery and adherence were planned to be monitored, with strategies potentially involving tracking user engagement and satisfaction. | The planned adherence and fidelity of the intervention were not detailed in the document. | The study planned to assess the effects of the intervention on coping strategies using pre- and post-intervention measures. | The intervention was planned to offer a meaningful legacy-making experience for families, with the process being easy to implement and cost-effective. | Planned to provide emotional support through creative expression, aiming to help parents process grief and maintain bonds with their child. | The study was planned to capture detailed qualitative data on the effects of memory-making from the parents' perspective, emphasizing the emotional and psychological benefits. | The study was planned to explore the therapeutic potential of HBRs in aiding parental bereavement and coping with chronic sorrow. |
| **HOW WELL (Actual)** | If intervention adherence or fidelity was assessed, describe the extent to which the intervention was delivered as planned. | Intervention delivered as planned, with high participation (78%) and retention (96%) rates, and positive feedback from children and parents. | As above, the study focused on perceptions and did not implement or assess an intervention's delivery. Since this study focused on exploring perceptions, the actual delivery and adherence to a developed intervention were not assessed. | Feedback from parents suggested the intervention was well-received and beneficial, indicating the intervention was delivered effectively with positive perceived benefits for children and families. | Positive feedback from parents suggested the intervention was well-received and beneficial, indicating effective delivery and perceived benefits for children and families. | The intervention acted 2015-2018. The intervention showed trends toward increasing the use of certain coping strategies among parents, although these were not statistically significant. | The study noted small effects on child procedural anxiety and perceived physical appearance, but these were not statistically significant. | The intervention showed small and non-significant effects on improving primary-control coping strategies and reducing disengagement coping strategies among participants. | The program was well-received, with all surveyed families recommending it to others facing similar end-of-life decisions. | Reported to be beneficial by participants, providing comfort, facilitating mourning, and helping to maintain an ongoing connection with the deceased child. | The activities were reported to be deeply meaningful and supportive by the participating parents, effectively aiding in their grieving process. | Effective in providing emotional support to parents, facilitating coping with grief, and creating a lasting memory of the child. |

**Supplemental File 5.** Details of the Mixed Methods Appraisal Tool (MMAT) evaluations

**1.MMAT Table for Qualitative Articles with Screening Questions**

| **ID** | **Study Reference** | **SQ1** | **SQ2** | **1.1** | **1.2** | **1.3** | **1.4** | **1.5** |
| --- | --- | --- | --- | --- | --- | --- | --- | --- |
| **1** | Akard et al 2018 | ✔ | ✔ | ✔ | ✔ | ✔ | ✔ | ✔ |
| **2** | Clarke and Connolly 2022 | ✔ | ✔ | ✔ | ✔ | ✔ | ✔ | ✔ |
| **3** | Schaefer et al 2020 | ✔ | ✔ | ✔ | ✔ | ✔ | ✔ | ✔ |
| **4** | Walden et al 2021 | ✔ | ✔ | ✔ | ✔ | ? | ✔ | ✔ |

- **Screening Question 1:** Are there clear research questions?
- **Screening Question 2:** Does the collected data address the research questions?
- **1.1** Is the qualitative approach appropriate to answer the research question?
- **1.2** Are the qualitative data collection methods adequate to address the research question?
- **1.3** Are the findings adequately derived from the data?
- **1.4** Is the interpretation of results sufficiently substantiated by data?
- **1.5** Is there coherence between qualitative data sources, collection, analysis, and interpretation?

**2.MMAT Table for Quantitative Randomized Control Trial (RCT) Articles with Screening Questions**

| **ID** | **Study Reference** | **SQ1** | **SQ2** | **2.1** | **2.2** | **2.3** | **2.4** | **2.5** |
| --- | --- | --- | --- | --- | --- | --- | --- | --- |
| **1** | Cho et al 2023 | ✔ | ✔ | ✔ | ✔ | ✔ | ✔ | ✔ |
| **2** | Terrah et al 2021 A | ✔ | ✔ | ✔ | ✔ | ✔ | ✔ | ✔ |
| **3** | Terrah et al 2021 B | ✔ | ✔ | ✔ | ✔ | ✔ | ✔ | ✔ |
| **4** | Terrah et al 2021 C | ✔ | ✔ | ✔ | ✔ | ✔ | ✔ | ✔ |
| **5** | Terrah et al 2015 | ✔ | ✔ | ✔ | ✔ | ✔ | ✔ | ✔ |
| **6** | Terrah et al 2020 | ✔ | ✔ | ✔ | ✔ | ✔ | ✔ | ✔ |

- **Screening Question 1:** Are there clear research questions?
- **Screening Question 2:** Do the collected data allow to address the research questions?
- **2.1** Is randomization appropriately performed?
- **2.2** Are the groups comparable at baseline?
- **2.3** Are there complete outcome data?
- **2.4** Are outcome assessors blinded to the intervention provided?
- **2.5** Did the participants adhere to the assigned intervention?

**3.MMAT Evaluation Quantitative Non-Randomized Studies with Screening Questions**

| **ID** | **Study Reference** | **SQ 1** | **SQ 2** | **3.1** | **3.2** | **3.3** | **3.4** | **3.5** |
| --- | --- | --- | --- | --- | --- | --- | --- | --- |
| **1** | **Andrews et al 2020** | ✔ | ✔ | ? | ✔ | ✔ | ✔ | ✔ |

Screening Question 1: Are there clear research questions?

Screening Question 2: Do the collected data allow to address the research questions?

3.1. Are the participants representative of the target population?

3.2. Are measurements appropriate regarding both the outcome and intervention (or exposure)?

3.3. Are there complete outcome data?

3.4. Are the confounders accounted for in the design and analysis?

3.5. Is the intervention (or exposure) administered as intended?
